# Laparoscopic sleeve gastrectomy in polysplenia syndrome/left isomerism: A case report

**DOI:** 10.1016/j.ijscr.2020.09.079

**Published:** 2020-09-16

**Authors:** Fatemah Jeragh, Ismaiel Aljazzaf, Haitham Al Khayyat

**Affiliations:** General Surgery Department, Mubarak Al-Kabeer Hospital, Ministry of Health, Kuwait

**Keywords:** Polysplenia syndrome, Left isomerism, Laparoscopic sleeve gastrectomy, Bariatric surgery, Morbid obesity

## Abstract

**Introduction:**

This is a case report that presents an unusual case of Polysplenia Syndrome (PSP) undergoing laparoscopic sleeve gastrectomy (LSG). This kind of presentation would be of interest to general surgeons who perform bariatric surgery.

**Presentation of case:**

The case presented is a 28-year-old female patient who was not a known case of Polysplenia Syndrome. During the patients’ pre-operative assessment, physical examination was done and a type of heterotaxy syndrome was suspected upon palpation of the stomach. Therefore, Computed Tomography (CT) imaging was performed which confirmed PSP. She underwent laparoscopic sleeve gastrectomy due to her morbid obesity, as her Body Mass Index (BMI) was 40.8 kg/m^2^. Post-operatively, water soluble contrast study was performed to rule out leak and the patient had uneventful follow-up.

**Discussion and conclusion:**

The surgical approach of such patients is somewhat similar to those with situs solitus; however, the performing surgeon must take into consideration the anatomical orientation. Proper assessment of such patients prior to surgery with history, full physical examination, and fitting imaging modalities is essential.

## Highlights

• A case of Polysplenia Syndrome (PSP) undergoing laparoscopic sleeve gastrectomy.
• It is a type of heterotaxy syndrome where there are multiple spleens congenitally, with left sided isomerism.
• The patient was not a known case of Polysplenia Syndrome, and it was considered during pre-operative assessment by palpating the abdomen.
• Significant CT findings include a right-sided stomach, complete intestinal malrotation, and a midline terminal ileum and cecum.
• During the surgery, the ports locations were changed to comply with the patients’ anatomy.

## 1 Introduction

Heterotaxy is defined as “an abnormality where the internal thoraco-abdominal organs demonstrate abnormal arrangement across the left-right axis”. Therefore, heterotaxy does not include situs solitus “the normal arrangement” nor situs inversus “the complete mirror-imaged arrangement”, but includes situs ambiguous “the ones in between” [1]. Polysplenia syndrome (PSP), also known as left isomerism, is a type of heterotaxy syndrome where there are multiple spleens congenitally, with left sided isomerism. This condition is known as left isomerism, and not right isomerism, due to the duplication of the left-sided structures. This condition is rare, even more than situs inversus. A precise incidence is not available due to the absence of severe congenital heart disease, therefore, most of the cases are found incidentally during imaging for unrelated conditions. It is predominantly seen in female patients, and was found to be higher in Asian Muslims due to inbreeding [2]. Polysplenia usually results from developmental failure of the usual asymmetry of the organs and is the usual form of situs ambiguous. In such condition, usually multiple spleens are seen but may have a single normal spleen, and some cases present as isolated reversal of splenic position. Although less common than Asplenia Syndrome (ASP), Polysplenia Syndrome patient have an increased risk of complex cardiac anomalies [3]. Other common features include inferior vena cava (IVC) interruption with azygos continuation, bilateral bilobed lungs, truncated/short pancreas or agenesis of dorsal pancreas, as well as intestinal malrotation, midline liver with biliary abnormalities, and an aorta that is located left of the midline [4,5].

Due to the high rates of obesity, the need of bariatric surgery has been increasing, especially in Kuwait, as it is considered one of the top 10 countries in the world in terms of obesity, with 42.8% of its’ population as obese. In addition, it has been found that the percentage of obesity (BMI of 30 kg/m^2^ and more) is higher in females than males, with 48% and 36% respectively [6]. Laparoscopic sleeve gastrectomy (LSG) is one of the widely performed bariatric surgeries due to its considerable safety and long-term results. It is important to present this case of Polysplenia syndrome/left isomerism, as it benefits other surgeons to well prepare themselves, in case of performing surgeries on such patients, in regards to expectations and complications.

In this paper, a case of a Polysplenia Syndrome patient with morbid obesity who underwent laparoscopic sleeve gastrectomy is presented, where diagnosis was made pre-operatively using a CT scan with intravenous contrast.

This case report has been reported in line with the SCARE criteria [7].

## 2 Case report

A 28-year-old female patient applied for an elective laparoscopic sleeve gastrectomy, she had a BMI of 40.8 kg/m^2^. (Weight: 98 kg, Height: 1.55 m). She is a known case of hypothyroidism on levothyroxine 50 mcg. She tried to lose weight using multiple diets, all of which have failed.

During the patients’ pre-operative assessment, full physical examination was performed. Upon palpating the abdomen, the stomach was felt on the right side instead of the usual left. This raised doubts about the presence of a type of heterotaxy.

Routine laboratory tests including complete blood count, renal and liver function test, coagulation profile, and thyroid function test were all performed, all were within normal ranges. Moreover, chest radiograph and electrocardiogram were both insignificant. She was also seen by the anesthesiologist and was cleared for surgery.

In regards to imaging, contrast study of the stomach and duodenum, using gastrograffin meal, was done which showed the stomach located on the right side of the abdomen, and jejunal loops on the left upper part of the abdomen, with no evidence gastro-esophageal reflux or hiatus hernia. After that, abdominal ultrasound was performed with limited visualization due to the patients obesity. A left sided enlarged liver with a normal gallbladder were the only findings. These findings has led to the need for a CT scan for proper visualization of the patients’ anatomy.

**Fig. 1:**
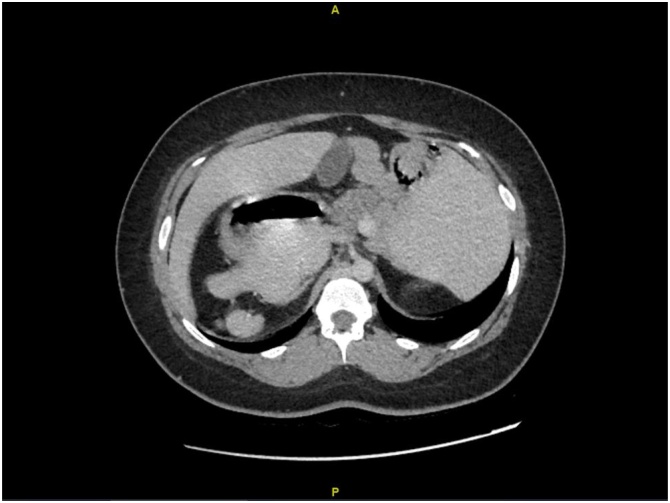
This is the patient’s CT scan in an axial plane, in which you can see a right sided stomach, and you can see the liver’s edges on both the right on left side.

**Fig. 2:**
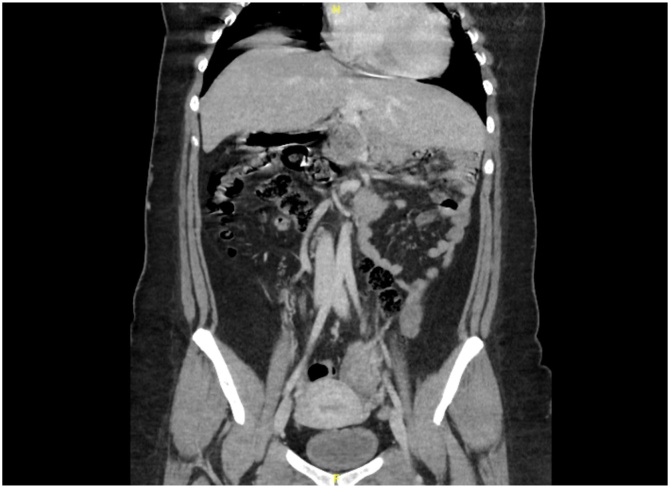
This is a coronal view of the CT scan showing the midline liver.

The CT abdomen with intravenous contrast was performed pre-operatively, where multiple significant findings were seen and diagnosis of Polysplenia Syndrome was confirmed. These findings include a right-sided stomach, a duodenum crossing anterior to mesenteric vessels, complete intestinal malrotation as the small bowels are on the left side and the large bowel on the right side, and the terminal ileum and cecum are midline. Other finding include, right sided splenules with a parent spleen, a midline liver, and infra-renal IVC with infra-hepatic interruption. The partially seen apex of the heart was at the left side. The gallbladder, adrenals, and urinary bladder showed no abnormality. The impression was that these features are suggestive of Polysplenia Syndrome/left isomerism (Fig. 1, Fig. 2).

**Fig. 3:**
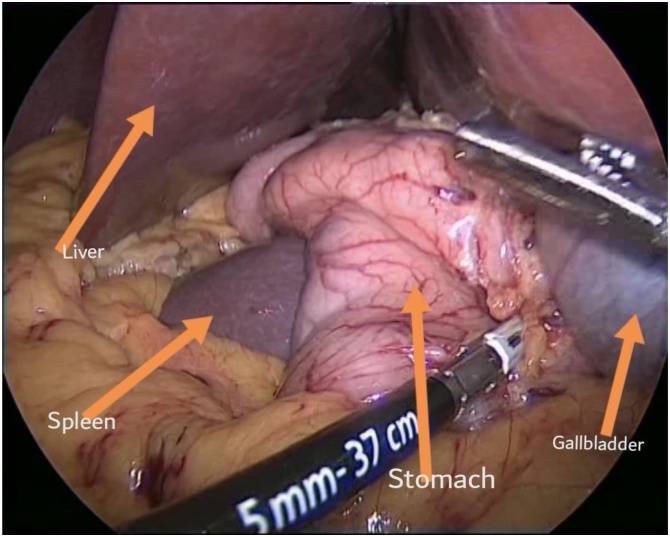
Here is an intraoperative view, in which the liver, spleen, stomach and gallbladder are all seen on the right side of the abdomen.

Laparoscopic sleeve gastrectomy was then performed by a bariatric surgeon under general anesthesia, with the patient in supine position. Starting with insufflation of the abdomen by a Veress needle at the right upper quadrant to create a pneumoperitoneum. Four ports were inserted under vision starting from the supraumbilical port; 11 mm, right and left midclavicular line; 5 and 15 mm respectively, and a 5 mm port at the epigastric area. The 15 mm port was inserted on the left to comply with the patients’ anatomy. The intraoperative findings include a large fatty midline liver, a right sided stomach, with a spleen posterior to it (Fig. 3). Dissection was started at the gastrocolic ligament at 6 cm from the incisura by using a 5 mm ultrasonic device (LigaSure). The dissection continued from the incisura to the angle of His, with sealing of the short gastric vessels. The posterior attachment with the stomach was then removed. Bougie size 38 was inserted up to the pylorus, then 2 blacks and 3 purples were inserted using a reinforced stapler device (Signia). The hemostasis was reinsured then methylene blue test was done, as it is regularly performed, showing no leak or twist. The resected stomach was removed from the 15 mm port. Closure of the 15 mm port fascia was done using Vicryl and Endoclose. The skin incisions were closed subcuticularly, using Monocryl.

The only difference in this approach from the one used in the situs solitus patients undergoing LSG is the location of the 15 mm port, as it was inserted on the left midclavicular line instead of the right.

**Fig. 4:**
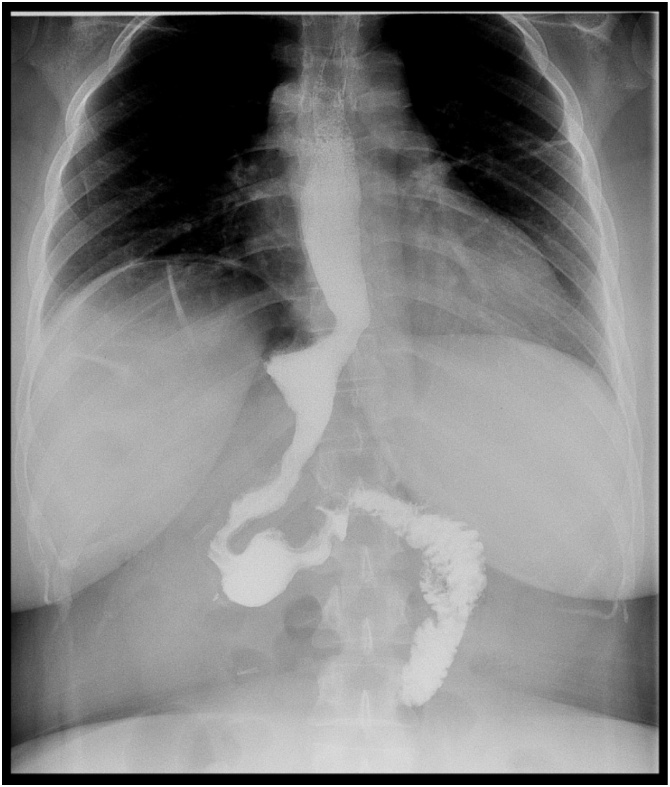
This is a water-soluble contrast study done one day post-operatively using gastrograffin meal showing no contrast leak.

**Fig. 5:**
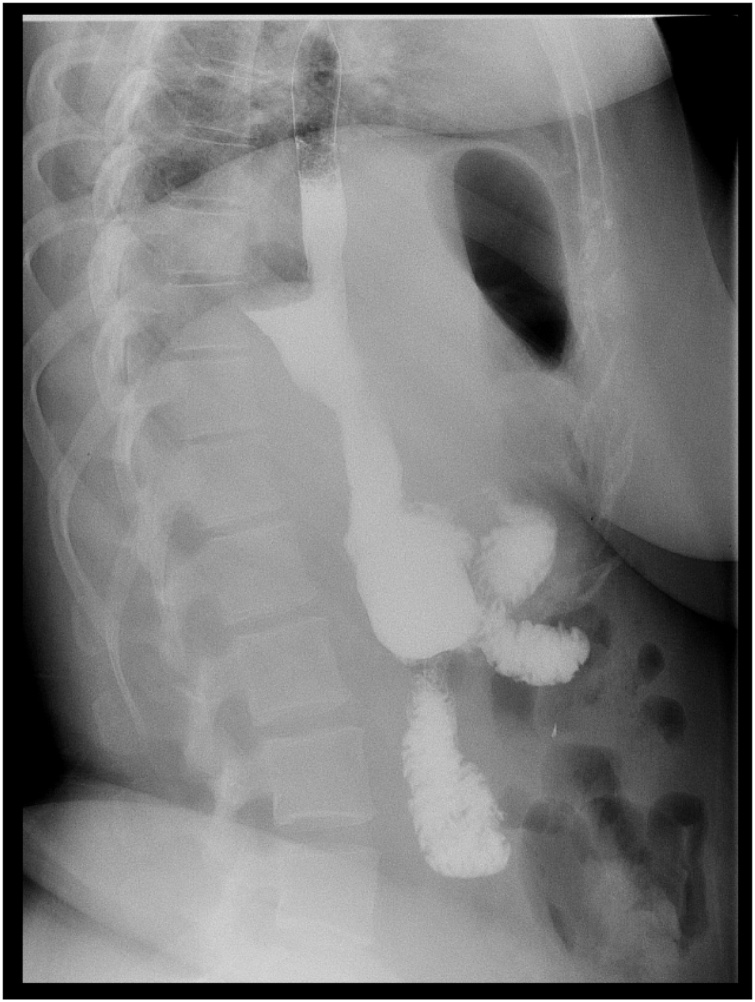
An axial view of the water-soluble contrast study done one day post-operatively.

Day one post-operatively, imaging was performed to rule out incision site leak using single water-soluble contrast study of the stomach and duodenum, using gastrograffin meal, during which, free flow of the oral contrast is noted through the esophagus into the neo-stomach with no evidence of contrast leakage. The stomach emptied well into the duodenum, showing no evidence of contrast leak post laparoscopic sleeve gastrectomy (Fig. 4, Fig. 5). This imaging is not regularly done; however, it was advised due to the patients’ anatomy.

The patient was discharged on post-operative day two, after drinking approximately 800 mL of clear fluids. She was not complaining of any nausea or vomiting. Her pain was tolerated with paracetamol and was improving gradually. She also passed urine, flatus, and stool normally. She was seen by a dietitian and consulted regarding the proper diet after LSG. She was encouraged to have a clear fluid diet of one to two Liters a day until her next follow up. Mobility and exercise were also recommended.

Two and four weeks after the surgery, she was seen in the out-patient clinic for a follow up. The patient was doing well, tolerating clear fluid diet with no complaints of nausea or vomiting. She did not complain of gastro-esophageal reflux either. Histopathology results were checked, and showed chronic inactive gastritis. She has lost 10 kg and her BMI decreased to 36.6 kg/m^2^. She drinks around 600 mL per day. She has been encourage to exercise daily, and to be followed up again in one month.

## 3 Discussion

As bariatric surgeries are considered as non-urgent medically important surgeries, this gives the surgeon the proper time for optimizing the patient prior to entering the operation theater. It is essential to accurately diagnose the patient to be able to plan surgical and interventional procedures without errors that might damage important structures. Moreover, locations of the used ports and any other incisions must be re-considered depending on the patients’ unique anatomy.

## 4 Conclusion

There are multiple anatomical variation that can be seen during a surgeons’ practice, therefore, it is essential that the surgeon knows how to approach and asses the patients’ anatomy pre-operatively. All surgeons must keep in mind the importance of pre-operative physical examination, even if the patient was not known to have any conditions or is completely asymptomatic.

In conclusion, bariatric surgery is highly manageable in patients with anatomical anomalies with proper pre-operative preparation in regards to physical examination, and fitting radiological imaging.

## Declaration of Competing Interest

The authors report no declarations of interest.

## Funding

The research did not receive any funding.

## Ethical approval

The study is exempt from ethical approval – observational case report.

## Consent

Written consent was acquired from the patient.

## Author’s contribution

Fatemah Jeragh – Writing the paper, and submission (Guarantor).

Ismaiel Aljazzaf – Study concept, and review.

Haitham Al Khayyat – Final review.

## Registration of research studies

N/A.

## Guarantor

Dr Fatemah Jeragh.

## References

[1] Jacobs J.P., Anderson R.H., Weinberg P.M. (2007). The nomenclature, definition and classification of cardiac structures in the setting of heterotaxy. Cardiol. Young.

[2] Rameshbabu C.S., Gupta K.K., Qasim M., Gupta O.P. (2015). Heterotaxy polysplenia syndrome in an adult with unique vascular anomalies: case report with review of literature. J. Radiol. Case Rep..

[3] Hacking C. (2020). Polysplenia syndrome. Radiopedia. https://radiopaedia.org/articles/polysplenia-syndrome-1?lang=us.

[4] Rose V., Izukawa T., Moës C.A. (1975). Syndromes of asplenia and polysplenia. A review of cardiac and non-cardiac malformations in 60 cases with special reference to diagnosis and prognosis. Br. Heart J..

[5] Ghosh S., Yarmish G., Godelman A., Haramati L.B., Spindola- Franco H. (2009). Anomalies of visceroatrial situs. AJR.

[6] ALNohair S. (2014). Obesity in gulf countries. Int. J. Health Sci..

[7] Agha R.A., Borrelli M.R., Farwana R., Koshy K., Fowler A., Orgill D.P., For the SCARE Group (2018). The SCARE 2018 statement: updating consensus Surgical CAse REport (SCARE) guidelines. Int. J. Surg..

